# Characterization of Load Components in Resistance Training Programs for Kidney Transplant Recipients: A Scoping Review

**DOI:** 10.3390/sports13050153

**Published:** 2025-05-19

**Authors:** Jhonatan C. Peña, Lilibeth Sánchez-Guette, Camilo Lombo, Edith Pinto, Carlos Collazos, Blanca Tovar, Diego A. Bonilla, Luis A. Cardozo, Luis Andres Tellez

**Affiliations:** 1 Research and Measurement Group in Sports Training, School of Heath and Sport Sciences, Fundación Universitaria del Área Andina, Bogotá 111061, Colombia; clombo3@estudiantes.areandina.edu.co (C.L.); lcardozo11@areandina.edu.co (L.A.C.); 2 Science and Technologies of Physical Activity and Sport, Universidad Manuela Beltran, Bogotá 110311, Colombia; 3 Faculty of Health Sciences, Universidad Simón Bolívar, Baranquilla 111321, Colombia; lilibeth.sanchez@unisimon.edu.co; 4 Asociación Colombiana de Deportistas Trasplantados (ACODET), Bogotá 111221, Colombia; acodetcolombia@gmail.com; 5 Vicerrectoría de Investigaciones, Grupo de Ciencias Básicas y Laboratorios, Universidad Manuela Beltrán, Bogotá 110311, Colombia; carlos.collazos@docentes.umb.edu.co; 6 Research Group GUANACO, School of Heath and Sport Sciences, Fundación Universitaria del Área Andina, Bogotá 111061, Colombia; btovar@areandina.edu.co; 7 Research Division, Dynamical Business and Science Society–DBSS International SAS, Bogotá 110311, Colombia; dabonilla@dbss.pro; 8 Hologenomiks Research Group, Department of Genetics, Physical Anthropology and Animal Physiology, University of the Basque Country (UPV/EHU), 48940 Leioa, Spain; 9 Research Group in Physical Activity, Sports and Health Sciences (GICAFS), Universidad de Córdoba, Monteria 230002, Colombia; 10 Research Group ESCALA, Corporación Unificada Nacional de Educación Superior, Bogotá 111311, Colombia; luis_tellez@cun.edu.co

**Keywords:** renal transplantation, kidney grafting, exercise, strength training

## Abstract

Resistance training (RT) has been shown to produce beneficial effects, including on quality of life, renal function, physical fitness, and survival rates in kidney transplant for 24 recipients. However, the optimal periodization of load components for this population remains unclear, as no consensus has been established. This study aimed to characterize the load components of RT programs in kidney transplant recipients. A scoping review was conducted following the Preferred Reporting Items for Systematic Reviews and Meta-Analyses Extension for Scoping Reviews (PRISMA-ScR). The literature search was performed up to October 2024 in MEDLINE/PubMed, the Web of Science Core Collection, SCOPUS, ScienceDirect, and SPORTDiscus. Only studies that included RT as part of the intervention were considered. The RT variables analyzed included intervention duration, weekly frequency, session duration, number and types of exercises, intensity, number of sets, rest time between sets, progressive overload, and execution velocity. A total of 645 studies were identified, of which 15 met the eligibility criteria and were selected for analysis. The primary strategy for intensity control was based on the percentage of one-repetition maximum (%1RM), with training zones ranging from 30% to 80%. The number of sets varied from two to eight, while repetitions ranged from 10 to 20. The rest intervals between sets lasted between one and five minutes. The most highly implemented type of resistance involved the use of dumbbells, body weight, and elastic bands. A high degree of heterogeneity was identified in load periodization parameters, highlighting a lack of consensus in exercise prescription for this population. However, this review established general criteria that can serve as a reference for exercise professionals to develop more structured and effective training programs.

## 1. Introduction

Kidney transplantation is the most effective strategy for treating individuals with end-stage renal disease, providing multiple benefits such as improved quality of life and increased survival rates [1,2]. However, as part of their post-surgical treatment, these patients receive immunosuppressive medications, which weaken the immune system and increase the risk of metabolic disturbances associated with the recurrence of non-communicable diseases [3,4,5]. Given this issue, it is essential for these individuals to adopt healthy lifestyle habits that counteract the adverse effects of these medications and contribute to better immune system function [6,7,8]. In this regard, previous research has established that obesity, metabolic syndrome development, physical inactivity, and poor dietary habits are among the risk factors associated with premature mortality, cardiac events, and graft loss [9,10].

One of the most effective strategies for improving specific health outcomes in this population, while simultaneously reducing the risk of metabolic diseases, is the incorporation of physical exercise into their daily routines [11,12,13,14,15]. A recent meta-analysis, including a total of sixteen randomized clinical trials with 827 patients, demonstrated that supervised physical exercise resulted in favorable changes in cardiorespiratory capacity, physical fitness, creatinine levels, quality of life, and high-density lipoprotein cholesterol (HDL-c) values [16]. However, the findings of this study suggest that due to variability in interventions, it is not possible to conclude which exercise modality is the most effective. It was only established that most successful studies proposed a frequency of three sessions per week, with a duration of 30 to 60 min per session, over a period of three to six months. Furthermore, it is suggested that the cardiorespiratory component should be programmed at 60% to 70% of maximum heart rate, while strength training should be performed twice a week [16]. Additionally, a recent clinical practice guideline on lifestyle in renal disease, specifically in transplant recipients, indicates that engaging in physical exercise improves survival rates in these patients [17]. However, regarding load periodization parameters, the guideline only generally mentions the importance of combining resistance and endurance training, without delving into specific components such as intensity, volume, recovery times, density, and execution velocity.

In the last decade, there has been a paradigm shift in how resistance training (RT) is programmed and dosed [18]. In this regard, various studies have aimed to standardize the correct methodology for periodizing all load components [19,20]. RT has been widely used as an intervention strategy for kidney transplant recipients, demonstrating positive effects on health. However, there is considerable heterogeneity in how these exercise programs are prescribed, making it difficult to establish an optimal model for load periodization and progression. Currently, no specific guidelines provide clear recommendations for structuring strength training programs in this population, posing a challenge for exercise professionals. Given the need for evidence-based guidelines to support the programming of strength training for kidney transplant recipients, this exploratory review aims to characterize the load components used in these training programs. By identifying patterns in exercise prescription and detecting gaps in the literature, this study seeks to provide valuable insights that can contribute to the development of more precise recommendations for professional practice.

## 2. Materials and Methods

### 2.1. Protocol and Registration

A scoping review was conducted following the reporting Preferred Reporting Items for Systematic Reviews and Meta-Analyses Extension for Scoping Reviews (PRISMA-ScR) [21]. The review protocol was registered on Open Science Framework https://osf.io/2bh9z/ (accessed on 2 May 2024)-The Preferred Reporting Items for Systematic Reviews and Meta-Analyses Extension for Scoping Reviews (PRISMA-ScR) checklist is presented in Supplementary Table S1.

### 2.2. Eligibility Criteria

This review included primary studies with clinical trial designs (randomized or non-randomized controlled studies) that evaluated the effect of RT programs, either supervised or unsupervised on any type of outcomes in kidney transplant recipients. RT programs featuring free weights (i.e., with or without external overload [weights, elastic band]) and machines were considered. Study interventions with other types of methodologies such as endurance training (in treadmill or bicycle and multi-component) were not included in the scope of this review.

### 2.3. Information Sources

The search for studies was carried out in the following databases: MEDLINE/PubMed, the Web of Science Core Collection, SCOPUS, Science Direct, and SPORTDiscus. The reference lists of the selected articles were also manually searched for additional literature (snowballing).

### 2.4. Search Strategy

The population, concept, and context (PCC) mnemonic was utilized for structuring our research question [22]: P (kidney transplant recipients), C (RT protocols), and C (detailed RT variables according to the National Strength and Conditioning Association [NSCA] recommendations: training methodology, exercise selection, sets, repetitions, intensity, load progression, rest interval between sets, execution velocity, weekly frequency, and duration of RT intervention [23,24,25]). The search strategy was composed of the keywords: (“resistance training” OR “resistance exercise” OR “strength training” OR “strength exercise” OR “weight exercise” OR “weight training” OR “exercise training”) AND (“kidney transplantation” OR “renal transplantation” OR “kidney transplant recipients” OR “kidney grafting”). The search strategy for each specific base is presented in Supplementary Table S2. The searches were carried out without applying restrictions by year of publications, but only articles written in the English language were included. Before submitting the manuscript, an updated bibliographic search was conducted to incorporate the most recent and relevant studies. As a result of this update, two additional studies were included in the analysis.

### 2.5. Method for Evidence Source Selection

Two research carried out the searches in the proposed databases and the identified studies were imported into EndNote^TM^ Basic to remove the duplicates. Subsequently, the authors (JP and CL) reviewed the full text to determine the inclusion of the study according to the eligibility criteria. A third reviewer (LC) analyzed disagreements and determined which studies were included in the review.

### 2.6. Data Charting Process and Items

The selected studies were analyzed independently by each researcher, extracting the following information in an Excel rubric. In studies reporting combined exercises (concurrent training), only the RT information was extracted:

- General information of the study: year of publication, design, sample size, age, sex and post-transplant time of the participants.
- Characteristics of the RT program: training methodology, exercise selection, sets, repetitions, intensity, load progression, rest interval between sets, execution velocity, weekly frequency, and duration.

### 2.7. Data Extraction and Analysis Process

A qualitative descriptive analysis of the training programs was carried out. The researchers extracted the information presented in each manuscript in the methodology sections. In cases where one of the proposed characteristics was not clearly specified in the text, it was recorded as “not reported”. At the end of this process, cross-checking of all the information retrieved was carried out. A third review by an experienced proofreader resolved the divergences.

## 3. Results

### 3.1. Selection of Sources of Evidence

**Figure 1:**
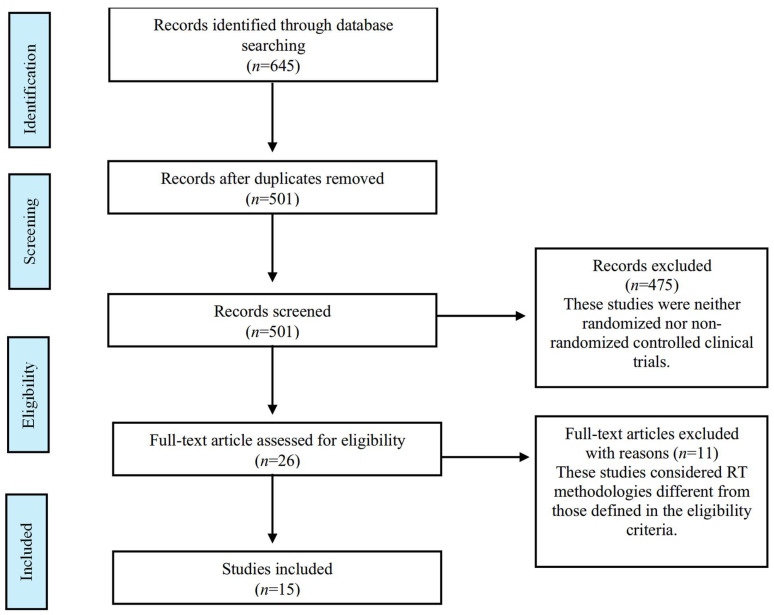
PRISMA flow diagram.

Initially, 645 studies were identified. After removing duplicates, 501 titles and abstracts were screened, resulting in 26 potentially eligible full texts. A total of 15 studies were included in this review. Figure 1 presents the PRISMA flow diagram of this study.

### 3.2. Characteristics of Sources of Evidence

Table 1 shows the characteristics of the selected studies. A total of 666 participants were included across all studies, with both men and women taking part in the 15 interventions [26,27,28,29,30,31,32,33,34,35,36,37,38,39,40]. The age of the experimental groups ranged from 27 to 56 years. The post-transplant time before initiating the physical exercise program was reported in only five studies with a mean of 57.6 months.

### 3.3. Synthesis of Results

Table 2 summarizes the exercise prescription details. Three studies focused on RT [27,32,38], two compared endurance and RT [34,37], and the remaining implemented concurrent training [26,28,29,30,31,33,35,36,39,40]. Training frequency ranged from three to five sessions per week, the intervention duration ranged from 2.5 to 12 months, and session length ranged from 30 to 90 min. Six studies used light dumbbells, body weight, and elastic bands [28,29,30,31,33,35], while four included machine-based exercises [27,37,38,40]. Four studies did not specify the exercise type [26,32,34,36]. The number of exercises per session ranged from 5 to 12.

Table 3 details the periodization characteristics. Intensity was primarily controlled using a percentage of one-repetition maximum (1RM) [26,32,35,36,37,38,40]. Other methods included OMNI scale [30], maximum heart rate (MHR) [28], and maximum repetitions [27]. Sets ranged from two to eight, repetitions from 10 to 20, and rest intervals from 1 to 5 min. Load progression was based on intensity, volume, or exercise complexity. Execution velocity was reported in one study [30] using self-perception.

## 4. Discussion

This review provides a comprehensive analysis of the characterization of load components in RT programs for kidney transplant recipients. Based on the analysis of thirteen studies, certain trends were identified; however, one of the main findings of this review was the high variability in exercise prescription and the lack of standardized criteria for load dosing.

Regarding the type of training, most of the reviewed studies suggest a concurrent training methodology [26,28,29,30,31,33,35,36,39,40]. This approach integrates RT as a fundamental component of the training session while complementing it with cardiorespiratory exercises. In fact, evidence from other populations indicates that concurrent training does not impair muscle hypertrophy or maximal strength development [41]. However, gains in explosive strength may be attenuated, particularly when cardiorespiratory exercise and RT are performed within the same session [42]. Moreover, concurrent training appears to be an effective exercise methodology for enhancing motivation and adherence, particularly among sedentary individuals with no prior experience in structured training programs [43,44,45]. This could explain its frequent implementation in the transplant population.

Load intensity is a critical variable in RT load dosing across different populations [46,47,48,49]. Among the studies included in this review, six used %1RM to control this variable [26,32,35,36,37,38,40], making it the most commonly employed indicator. However, the reported training intensities ranged from 30% to 80% of 1RM, highlighting a lack of conceptual clarity regarding the optimal intensity range for kidney transplant recipients.

Regarding volume, the number of exercises per session ranged from five to eight, the number of sets varied between two and eight, and repetitions ranged from 10 to 20. Similarly to intensity, these broad ranges reflect the absence of conclusive scientific evidence establishing precise training volumes to induce specific chronic adaptations, as has been determined for other populations [50,51]. Similarly, it was observed that rest intervals between sets is a fundamental variable for specific adaptations such as hypertrophy development [52] were not reported in nine of the reviewed studies [26,28,29,32,33,34,35,36,39]. This suggests that the importance of this variable has been overlooked when designing training protocols for transplant populations.

Among the load components, execution velocity has received the least attention in this population, with only one study reporting its use [30]. In contrast, research in other populations (i.e., older adults) has demonstrated that high-speed resistance training is effective for improving overall health outcomes [53]. It is worth mentioning that movement velocity is an accurate approach for prescribing RT, offering multiple advantages such as controlling intensity from the first repetition of a set [54,55,56] and regulating volume based on velocity loss [20,57]. Controlling this variable eliminates many of the inconsistencies associated with traditional RT methods of RT [18]. If a linear position transducer is not available, perceived exertion and movement velocity scales serve as valid, economical, and practical tools for assessing RT load progression and complementing other training monitoring variables, although familiarization remains key [58].

Regarding exercise selection, six of the reviewed studies recommended the use of light dumbbells, body weight exercises, and elastic bands [28,29,30,31,33,34]. A possible rationale for this recommendation is the ease of execution, particularly for individuals with limited RT experience, a common characteristic in this population.

One of the main limitations of this study is that only 15 out of the 501 initially identified studies were included. While the strict eligibility criteria ensured the quality of the analyzed studies, they may have excluded some training programs developed for this population. It is important to note that the scope of this review was limited to the load components of strength training programs; the effects of these interventions on the outcome variables assessed in each study were not analyzed. Additionally, certain strength training methodologies, such as suspension exercises, were not considered.

Considering the absence of standardized criteria for periodizing load components in kidney transplant recipients, Table 4 summarizes the most frequently reported elements in the reviewed studies. These elements can serve as a foundation for exercise professionals in designing training programs for this population.

### Future Directions

One of the most significant insights derived from this review is the recognition that, while a substantial body of scientific evidence supports the beneficial effects of structured exercise programs on various health outcomes, notable methodological limitations continue to persist within the design and implementation of these training protocols. These shortcomings highlight the need for a more rigorous and standardized approach to exercise prescription, particularly in clinical populations where individualized interventions are crucial. In this regard, fostering interdisciplinary collaboration among nephrologists, sports medicine physicians, and exercise professionals presents a promising avenue for addressing these challenges. By integrating their respective expertise, these specialists could contribute to the development of more precise, evidence-based, and systematically structured training regimens tailored to the specific physiological and medical needs of patients. Such an approach would not only enhance the efficacy of exercise interventions, but also ensure their safety and long-term adherence.

The heterogeneity of the implemented programs hinders the establishment of specific and detailed parameters for load prescription. However, the common elements identified can serve as a foundation for exercise professionals to design more structured and effective training plans. Future research should focus on developing more rigorous protocols supported by robust methodological frameworks that include well-defined exercise parameters, appropriate control conditions, and standardized outcome measures. Additionally, it is essential to determine the optimal exercise modalities, intensities, and durations that maximize therapeutic benefits in patients with various health conditions. Strengthening the scientific foundation in this field will facilitate the integration of structured exercise as a fundamental component of clinical care, enhancing both the effectiveness of interventions and the overall health and well-being of patients.

## 5. Conclusions

This scoping review described the general characteristics of the load components in RT programs for kidney transplant recipients. The findings highlight a lack of standardized criteria in the prescription of fundamental periodization variables; however, several recommendations based on available evidence are given regarding intensity, volume, and load progression. Additionally, movement velocity remains an overlooked component in the design of strength training programs for this population.

## Figures and Tables

**Table 1:** General characteristics of the studies.

Author	Research Design	Sample Size	Sex	Age Experimental Group (Years)	Post-Transplant Time (Experimental Group)
Roi et al., 2018 [26]	NRCT	EG:52 CG:47	F/M	47 ± 12	5.1 ± 2.1 (years)
Hernández et al., 2021 [27]	RCT	EG:8 CG:8	F/M	49.7 ± 9.6	115 ± 54 (months)
Hemmati et al., 2022 [28]	RCT	EG:13 CG:10	F/M	32.9 ± 9.81	NR
Michou et al., 2023 [29]	RCT	EG:13 CG:12	F/M	54.9 ± 9.9	NR
Lima et al., 2019 [30]	RCT	EG:7 CG: 15	F/M	54 ± 3	4 ± 1.8 (years)
Michou et al., 2022 [31]	RCT	EG:11 CG:10	F/M	52.9 ± 9.5	47.4 ± 18.3 (months)
Eatemadololama et al., 2017 [32]	NRCT	EG:12 CG:12	NR	27.4 ± 17.3	NR
Zhang et al., 2023 [33]	RCT	EG:53 CG:53	F/M	43.16 ± 10.76	NR
O’Connor et al., 2017 [34]	RCT	EGRT:13 EGET:13 CG:20	F/M	54.6 ± 10.6	NR
Senthil et al., 2020 [35]	RCT	EG:53 CG:51	F/M	36.2 ± 8.6	NR
Riess et al., 2014 [36]	RCT	EG:16 CG:15	F/M	56.9 ± 12.2	NR
Greenwood et al., 2015 [37]	RCT	EGRT:20 EGET:20 CG:20	F/M	54.6 ± 10.6	NR
Karelis et al., 2016 [38]	RCT	EG:10 CG:10	F/M	45.3 ± 14	2 to 18 (months)
Crepaldi et al., 2024 [39]	RCT	EG:8 CG:7	F/M	52 ± 10	NR
Billany, 2025 [40]	RCT	EG:25 CG:25	F/M	49 ± 13	NR

RCT: randomized controlled trial, NRCT: non-randomized controlled trial, F: female, M: male, NR: not reported, EG: experimental group, CG: control group, EGRT: experimental group resistance training, EGET: experimental group endurance training.

**Table 2:** General components of training program load.

Study	Training Type	Frequency (Days of Week)	Duration (Months)	Session Duration (Minutes)	Number of Exercises	Types of Exercises
[26]	CT	3	12	60	5	NR
[27]	RT	2	2.5	60	NR	Exercises involving the upper and lower limb such as leg press, rowing pulley, leg curl, flymachine, machine calf raises, knee extension and core work.
[28]	CT	3	3	60 a 90	NR	Exercises for upper and lower extremities and abdominal muscles using free weights or body weight.
[29]	CT(SP)	3	6	60 a 70	3 a 6	Dynamic muscle-strengthening exercises.
[30]	CT	3	3	35	5	Exercises with free weights.
[31]	CT (SP)	3	6	60 a 90	6	Exercise with body weight.
[32]	RT	2	3	60	NR	NR
[33]	CT	2	6	30	2 a 3	Exercises with light dumbbells, body weight and elastic bands.
[34]	ET and RT	2	3	NR	NR	NR
[35]	CT	2	3	NR	NR	Exercises with the use of gravity and own body weight in phase 1 and exercises were performed for quadriceps with external loads for phase 2.
[36]	CT	5	3	NR	NR	NR
[37]	ET and RT	1	3	60	8	Bench press, latissimus pulldown, bicep curl, triceps pull down, leg press, knee extension, hamstring curl, and calf raises
[38]	RS	3	2.5	45 a 60	7	Leg press, chest press, lat pulldowns, shoulder press, arm curls, triceps extensions, and sit-ups
[39]	CT	3	6	60	5	Leg extension, pectoral machine, lat machine, leg curl, and abductor machine.
[40]	CT (SP)	5	3	60	12	Squat, hip abduction, lunge, calf-raise, side-lunge, bicep-curl, bent-over row, reverse-fly, lateral-raise, chest-press, side-bends, and standing trunk rotation.

RT: resistance training, ET: endurance training, CT: concurrent training, SP: without face-to-face support, NR: not reported.

**Table 3:** Specific components of training program load.

Study	Intensity	Sets	Repetitions	Rest Time Between Sets (Minutes)	Progressive Load	Velocity
[26]	35% 1RM	2	20	NR	An increase in load is indicated at 6 months but no further information is given.	NR
[27]	10 RM	3	10	1	Increased volume was progressively throughout the training program coming to complete four sets of 10-RM.	NR
[28]	40 al 65% MHR	NR	NR	NR	An increase in intensity is indicated based on the response of each participant but the way in which it was performed is not mentioned.	NR
[29]	NR	2	8 a 10	NR	The load was adjusted with changes in positions during the development of the exercises and including balls, bands, and dumbbells.	NR
[30]	6 A 7 (OMNI)	2	10	1	NR	Controlled by self-perception.
[31]	NR	8	10	1	NR	NR
[32]	50% 1RM	NR	10 a 15	NR	An increase of 5 to 10% of 1RM is indicated but the moment in which it was performed is not specified.	NR
[33]	NR	NR	10	NR	It is mentioned that first the number of repetitions was increased, then the number of sets was increased from one to two, and finally the resistance levels of dumbbells or elastic bands were increased.	NR
[34]	NR	NR	NR	NR	NR	NR
[35]	50 al 85% 1RM	NR	10	NR	Resistance was increased in a graded manner at a rate of 5% to 10% of the previous load.	NR
[36]	50% 1RM	2	10	NR	The intensity increased by 5–10% when two sets of 15 repetitions were performed while adhering to strict technique.	NR
[37]	80% 1RM	1 a 3	8 a 10	3	The one-repetition maximum was reassessed monthly, and the program was adjusted accordingly.	NR
[38]	80% 1RM	3	10	1 a 5	Weekly increase based on ability to maintain prescribed number of repetitions	NR
[39]	NR	NR	NR	NR	It is indicated that the progression ranged between 40 and 50% and 65–75% of a person’s maximum exercise capacity, but it is not indicated with which load component these percentages were estimated.	NR
[40]	60%1RM	2	10	1	The progression of the load was achieved by increasing the number of series per session.	NR

1RM: one-repetition maximum, MHR: maximum heart rate, OMNI: Perceived Exertion Scale for Resistance Exercise, NR: no reported.

**Table 4:** General recommendations for prescribing RT for kidney transplant recipients.

Load Component	Recommendation for This Component
Training Type 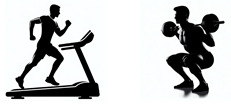	RT might be complemented with concurrent training. Adherence is crucial.
Frecuency and session duration 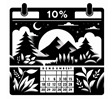	The recommended frequency is at least twice per week. Each session should last a minimum of 30 min.
Types of Exercises 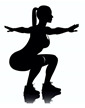	Bodyweight exercises and light dumbbells are the most recommended modalities.
Number of exercises 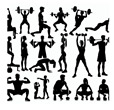	The number of exercises per session ranges from five to eight.
Intensity of load 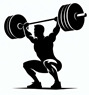	Intensity can be regulated as a percentage of 1RM, with work ranges varying between 30% and 80% of 1RM.
Training volume 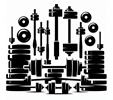	Training volume includes two to eight sets per exercise, with repetitions ranging from 10 to 20 per set.
Rest time between sets 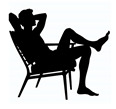	Rest intervals between sets vary between one and five minutes.
Progressive load 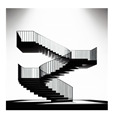	Load progression is achieved by increasing the intensity, volume, or technical difficulty of the exercises.
Execution velocity 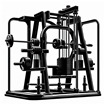	Execution velocity of movements has not been extensively reported in this patient population.

## Data Availability

The original contributions presented in the study are included in the article; further inquiries can be directed to the corresponding author.

## Supplementary Materials

The following supporting information can be downloaded at: https://www.mdpi.com/article/10.3390/sports13050153/s1, Table S1. Preferred Reporting Items for Systematic Reviews and Meta-Analyses Extension for Scoping Reviews (PRISMA-ScR) Checklist; Table S2. Detailed search strategy in each specific base.

## Abbreviations

The following abbreviations are used in this manuscript:

RT	Resistance training
1RM	One-repetition maximum
OMNI	Perceived Exertion Scale for Resistance Exercise
MHR	Maximum heart rate
